# Hudson’s Tooth Paste

**Published:** 1863-01

**Authors:** 


### HUDSON'S TOOTH PASTE

Is an article that is likely to meet the wants of the public. We have used the paste, and like it. We have *not analyzed* it—could n't afford the time, but have not been led, by the slight examination we were able to give it, to suspect the presence of anything deletarious. The portrait on the label is, perhaps, in good taste; for Mr. H. is rather good looking. The "puffs" are too well laid on; but as the proprietor is a *non-professional*, we can excuse him if he only keeps us supplied with a good article. A perverted public taste impels him in this direction. People want everything puffed. They do n't even buy *maple sugar* from the candy man on the cars till they see, by the label, that it is "good for coughs, colds, etc." They do n't like sugar; but as they have a bad cough, and are threatened with *etcetera*, they will try and "worry down" a little of the *medicine*. So if friend H., by puffing a *little*, can induce the public to keep their teeth clean, he will be a benefactor to the race. W.

